# Efficient and interpretable maximal frequent fuzzy pattern mining with multi phase pruning and ternary search

**DOI:** 10.1038/s41598-026-57896-2

**Published:** 2026-06-13

**Authors:** Khalil Al-Wagih, Mukhtar Abdulmomen Abdullah, Ebrahim Mohammed Senan

**Affiliations:** 1 Department of Computer Science, Faculty of Computer Science & Informatics, Thamar University, Dhamar, Yemen; 2 Department of Information Systems, Faculty of Computer Science and Information Technology, Al-Razi University, Sana’a, Yemen; 3 Department of Computer Science, Faculty of Applied Sciences, Hajjah University, Hajjah, Yemen

**Keywords:** Maximal Frequent Pattern Mining, Pattern Explosion, Computational Efficiency, Knowledge Discovery, Data Mining Algorithms, Fuzzy Membership Functions, Search Space Pruning, Computational biology and bioinformatics, Engineering, Mathematics and computing

## Abstract

The exponential growth of quantitative data across various domains has intensified the need for efficient pattern mining techniques that can handle numerical uncertainty while maintaining interpretability. Traditional fuzzy frequent pattern mining algorithms suffer from pattern explosion in dense datasets, generating overwhelming numbers of redundant patterns that hinder practical analysis. This study introduces a novel Maximal Frequent Fuzzy Pattern Mining (MFPM) framework that integrates fuzzy set theory with maximal pattern representation to address these limitations. The proposed methodology employs a multi-phase approach that incorporates aggressive pruning strategies, including maximum cardinality selection and early termination, to reduce the dimensionality of the search space. Evaluation on three datasets (Chess, Connect and Mushroom) demonstrates consistent gains in both effectiveness and efficiency. Time-wise, MFPM accelerates discovery where classical algorithms are slowest: dense regimes and permissive supports. A ternary search algorithm efficiently identifies the longest patterns, while an Anti-Apriori strategy with superset pruning ensures the extraction of only non-redundant maximal patterns. Experimental evaluation on benchmark datasets demonstrates remarkable effectiveness, achieving up to 94.97% pattern reduction compared to traditional FTDA algorithms while maintaining equivalent knowledge representation. Computational efficiency improved by over 65% in challenging low-support scenarios. The framework generates concise, semantically interpretable patterns that capture the most significant relationships in quantitative data, facilitating informed decision-making across diverse application domains, including healthcare analytics, business intelligence, and web usage mining.

## Introduction

The digital age is characterized by the generation of vast amounts of data from numerous sources, including social networks, financial systems, medical sensors, and Internet of Things (IoT) devices^1^. The resulting phenomenon, now referred to as “big data,” presents both risks and opportunities^2^. The ability to analyze those large-scale datasets and extract useful information is essential for governments, research institutions, and companies to enhance service, improve efficiency, and foster^3^. The volume, velocity, and variety of data produced today often exceed the capabilities of traditional statistical methods, requiring advanced computational techniques^4^.

The notion of Knowledge Discovery in Databases (or KDD) has emerged in response to this shift – KDD is a systematic and iterative process for converting raw data into actionable knowledge^5^. The KDD pipeline includes data cleaning, data integration, data selection, and data transformation; during the final step, the knowledge of data mining reveals hidden patterns. Data mining is a data analysis step that employs extensive, specialized algorithms to discover implicit yet valuable relationships within large datasets^6^.

Frequent Pattern Mining is a crucial method in data mining that identifies sets of items that frequently appear together in a dataset, with a frequency exceeding a predefined threshold value, known as the minimum support (minsup)^7^. Frequent Patterns (FPs) identify inherent relationships within the data and apply to a range of problems, including bioinformatics, healthcare, cybersecurity, and marketing. Additionally, FPM is a crucial step in building more sophisticated data mining tasks, such as association rule mining and sequential pattern mining.

Older algorithms, such as Apriori, introduced the first FPM algorithms, which typically employed a level wise, breadth-first search mechanism based on the anti-monotonic property to identify all items that frequently occurred together^8^. These and other early approaches had significant computational drawbacks, including the need for multiple scans of the database and the generation of an overwhelming number of candidates^9^. The FP-Growth algorithm addressed these drawbacks by introducing a pattern-growth method that utilized a tree structure (i.e., the FP-Tree) to identify frequent patterns without generating candidates. However, the ongoing problem of pattern explosion in dense datasets or at low minsup thresholds typically generates an unmanageable number of FPs, which are computationally intensive and confusing for the analyst, thus negating the purpose of extracting the knowledge^10^.

To address this, research has shifted toward compressed representations of FPs, notably Closed Frequent Patterns (CFPs)^11^ and Maximal Frequent Patterns (MFPs)^12^. MFPs, which have no frequent supersets, provide the most compact representation, as all other FPs can be derived from them. This maximality property offers substantial benefits, including reduced storage needs, lower computational costs, and a concise, non-redundant summary of the most significant patterns, greatly enhancing interpretability for practical applications^13^.

A significant drawback of traditional FPM algorithms, including those for MFPs, is their design for binary data, where items are merely present or absent. However, real-world data often includes quantitative attributes such as age, income, or temperature. Applying binary mining to such data requires discretization into crisp intervals, which introduces the “sharp boundary problem.” Minute value changes can shift an item into a different interval, producing arbitrary and unstable patterns that lack robustness and intuitive meaning^14^.

Fuzzy set theory, introduced by Lotfi Zadeh, provides an elegant solution by allowing for partial membership. A quantitative value can belong to multiple fuzzy linguistic terms (e.g., “Low,” “Medium,” “High”) with varying degrees of membership between 0 and 1. This capacity to model uncertainty and graduality makes fuzzy logic highly effective. Converting quantitative data into fuzzy datasets creates a representation that is computationally feasible and semantically rich, aligning with human reasoning.

The fusion of fuzzy set theory with FPM has led to Fuzzy Frequent Pattern Mining (F´PM), which extracts Frequent Fuzzy Patterns (F´Ps) based on a minimum fuzzy support (f_minsup_). While many F´PM algorithms adapt Apriori or FP-Growth to the fuzzy domain, they inherit a critical weakness: the goal of finding all F´Ps. Consequently, they remain vulnerable to pattern explosion in dense quantitative datasets. The combination of multiple fuzzy terms and the downward closure property can yield an intractable number of patterns, consume excessive resources, and generate output too voluminous for practical use^15^. This research addresses this challenge by proposing a novel approach for mining Maximal Frequent Fuzzy Patterns (MF′Ps) from dense quantitative data. The proposed MFPM methodology synergistically combines the uncertainty-handling prowess of fuzzy set theory with the compactness of maximal pattern representation.

Despite advances in recurring pattern extraction, significant challenges remain when dealing with dense, quantitative, and uncertain data. Methods based on the Apriori algorithm often suffer from candidate bloat and redundant database scans. While the FP-Growth algorithm reduces candidate generation, it still focuses on detecting clusters of recurring patterns rather than on maximal fuzzy representations. Fuzzy recurring pattern extraction can handle quantitative uncertainty, but many approaches still generate excessively fuzzy outputs. Traditional maximal recurring pattern extraction reduces redundancy but is primarily designed for clear or binary transactional data.

The proposed MFPM framework addresses this gap by integrating fuzzy linguistic representation, maximal pruning, triple-search-based longest-pattern detection, and top-down maximal-pattern extraction using the Anti-Apriori algorithm. Therefore, the novelty of this study lies not in fuzzy logic or maximal pattern extraction separately, but in integrating these mechanisms into a unified, scalable, and interpretable framework for dense quantitative datasets.

By combining these components, MFPM maintains meaningful linguistic relationships while minimizing redundant pattern output, computational cost, and interpretation complexity.

The principal contributions of this study are as follows:

- Novel MFPM Framework: This study proposes an integrated framework that combines fuzzy set theory with maximal frequent pattern mining to handle uncertainty in quantitative data and generate compact fuzzy pattern representations.
- Early Search-Space Reduction: The proposed method introduces an early-stage maximum-cardinality pruning mechanism that retains the most representative fuzzy term for each attribute, thereby reducing the fuzzy search space and memory requirements.
- Efficient Longest Pattern Discovery: A ternary-search-based strategy is employed to identify the length of the longest frequent fuzzy pattern more efficiently than sequential exploration, reducing the search effort within this stage from linear to logarithmic behavior.
- Top-Down Maximal Pattern Mining: The framework applies an Anti-Apriori long-to-short search strategy combined with superset pruning to directly extract MFPs and reduce redundant candidate evaluation.
- Compact and Interpretable Output: The proposed method produces a compact set of MFPs, which reduces output redundancy while preserving meaningful linguistic relationships for decision-support purposes.
- Scalable and Generalizable Design: The modular design of the framework supports its adaptation to various quantitative data mining tasks, particularly in domains that require uncertainty handling, pattern compactness, and interpretability.

## Related work

In direct response to the reviewer’s request for comparison with maximal fuzzy, closed fuzzy, and fuzzy FP-Growth-based approaches, we conducted a focused comparative analysis of recent studies published between 2023 and 2026 that address redundancy reduction, uncertainty handling, and scalability in frequent pattern mining. Although substantial progress has been achieved, existing approaches still exhibit methodological limitations when applied to dense quantitative datasets. Pattern-growth approaches such as the Fault-Tolerant Frequent Pattern Tree (FT-FP-tree) framework proposed by Bashir et al.^16^ improved mining efficiency by avoiding Apriori-style candidate explosion and reducing repeated database scans. However, the framework primarily targets fault-tolerant maximal frequent itemsets in binary transactional environments and does not incorporate fuzzy semantic representation for quantitative uncertainty. Consequently, gradual linguistic relationships between numerical values cannot be modeled effectively.

Closed-pattern mining methods introduced by Jeya Sutha et al.^17^ and Elhady et al.^18^ improved runtime and storage efficiency through multiprocessing and fuzzy closed-itemset reduction strategies. Nevertheless, closed pattern mining preserves all closed patterns and their support information, which may still generate large volumes of patterns in dense datasets and reduce interpretability. In contrast, maximal pattern mining provides a more compact representation by retaining only the longest representative frequent patterns, although without preserving the support of all subsets explicitly. Therefore, while closed fuzzy mining reduces redundancy, it does not fully address the pattern explosion problem in high-dimensional fuzzy datasets.

Beyond maximal and closed pattern mining, several fuzzy Frequent Pattern Growth (FP-Growth) variants have focused on computational efficiency. Saikaew et al.^19^ improved FP-Growth through adaptive support thresholding, while Kumar et al.^20^ proposed a temporal three-dimensional fuzzy FP-tree to reduce memory usage and processing time. Similarly, Jeya Sutha et al.^21^ introduced a hash-based Type-2 fuzzy mining algorithm with efficient pruning mechanisms. Despite these improvements, these approaches continue to generate extensive frequent-pattern spaces because they focus on discovering all fuzzy frequent patterns rather than directly extracting maximal fuzzy representations. As a result, redundancy and output explosion remain significant limitations.

Wan et al.^22^ improved scalability and input/output (I/O) efficiency through prefix-partitioning and top-k mining strategies, whereas Mudumba et al.^23^ investigated distributed association rule mining across multiple data sources. Sadriddinov et al.^24^ explored maximal biclique discovery using Formal Concept Analysis (FCA), and Dogan et al.^25^ incorporated fuzzy theory into profit-supported association rule mining. Although these studies contributed to efficiency, distributed mining, and profit-oriented rule extraction, they do not integrate fuzzy linguistic modeling with maximal frequent fuzzy pattern extraction for quantitative transactional data.

Zhang et al.^26^ emphasize the importance of considering seasonal and periodic characteristics during the mining process, proposing a new model of seasonal-periodic subgraphs, complete with a novel naming scheme: maximal σ-periodic Ω-seasonal k-subgraphs. Their focus is on developing algorithms for efficiently discovering all maximal seasonal-periodic patterns. Regarding fuzzy pattern mining, Wu et al.^27^ introduced a fuzzy high-utility pattern mining algorithm along with its implementation in a parallel and distributed Hadoop framework. Their framework utilizes fuzzy-set theory in conjunction with MapReduce techniques to enhance mining patterns. However, they do say that high utility itemset mining is similar to frequent itemset mining in that both approaches rely on a binary threshold-based approach. Similarly, Cui et al.^28^ propose a fuzzy-based algorithm named FRI-Miner, with the specific purpose of mining for rare itemsets. The importance of fuzzy theory for infrequent patterns is emphasized to discover interesting and useful patterns, thereby expanding the field of fuzzy-pattern mining to help identify what could be classified as infrequent patterns. Fournier-Viger et al.^29^ elaborated on the notion of periodicity in pattern mining by introducing the notion of discovering local periodic patterns (LPPs) in sequences of events or transactions. The system is linked to methods for capturing temporal patterns in data. Temporal pattern mining, and sequential pattern mining also relate to the analysis of time series data. Wu et al.^30^ proposed OPP-Miner, an order-preserving sequential pattern mining algorithm, which discovers order-preserving patterns based on the relative ordering of measurements in time series data. The advantage here is that patterns are more meaningful because they can be order-preserving and allow us to see temporal dynamics. Additionally, Li et al.^31^ introduced MCoR-Miner, which focuses on maximal co-occurrence non-overlapping sequential rules, extracting sequential rules by reducing redundancy and improving the performance of recommendations. In regard to graph-based pattern mining, Nguyen et al.^32^, presented pmMine, a progressive framework for maximal frequent subgraph mining (MFSM). Their approach uses isomorphic graph embedding to simplify some of the computational complexity of subgraph isomorphism by providing approximate solutions in large graphs. Kumar et al.^33^made progress in fuzzy temporal data mining with their development of an improved fuzzy FP-growth algorithm. This algorithm aimed to enhance processing time and reduce memory consumption, while maintaining high accuracy, highlighting a continued focus on improving the efficiency of fuzzy pattern mining algorithms, particularly in temporal data settings. In a similar fashion, Zhou et. al^34^. implemented a fuzzy regional co-location pattern mining framework using maximal fuzzy grid cliques and density peak clustering. In this study, the researchers applied a maximal fuzzy grid clique property to identify probable regional patterns, indicating significant clustering, and showcased the application of maximal fuzzy structures to spatial data mining.

The comprehensive review of existing literature reveals a persistent and significant challenge: the efficient discovery of non-redundant, interpretable patterns from dense quantitative datasets. While previous research has made substantial progress in maximal pattern mining for binary data and fuzzy pattern mining for quantitative data, these two streams have largely progressed in parallel. The integration of maximality principles with fuzzy frequent pattern mining remains underexplored, with only isolated attempts that exhibit critical limitations. This represents a substantial methodological gap, as zero values often carry significant semantic meaning in real-world applications, such as representing missing purchases in market basket analysis or absent symptoms in medical diagnostics.

In contrast, the proposed MFPM framework introduces an integrated methodology that combines: (i) fuzzy linguistic transformation using triangular and trapezoidal membership functions (Sect. 3.2), (ii) early maximum-cardinality pruning for fuzzy search-space reduction (Sect. 3.3), (iii) ternary-search-based longest-pattern discovery for efficient maximal exploration (Sect. 3.5), and (iv) Anti-Apriori top-down maximal mining with superset pruning (Sect. 3.6). The novelty of MFPM does not arise from fuzzy logic or maximal mining individually, but from the methodological integration of these complementary mechanisms within a unified framework. Consequently, MFPM addresses key limitations related to redundancy, interpretability, and pattern explosion in existing maximal, closed, and fuzzy FP-Growth-based approaches. This effectiveness is further demonstrated by the substantial reduction in output patterns achieved by MFPM.

## Methodology

**Fig. 1:**
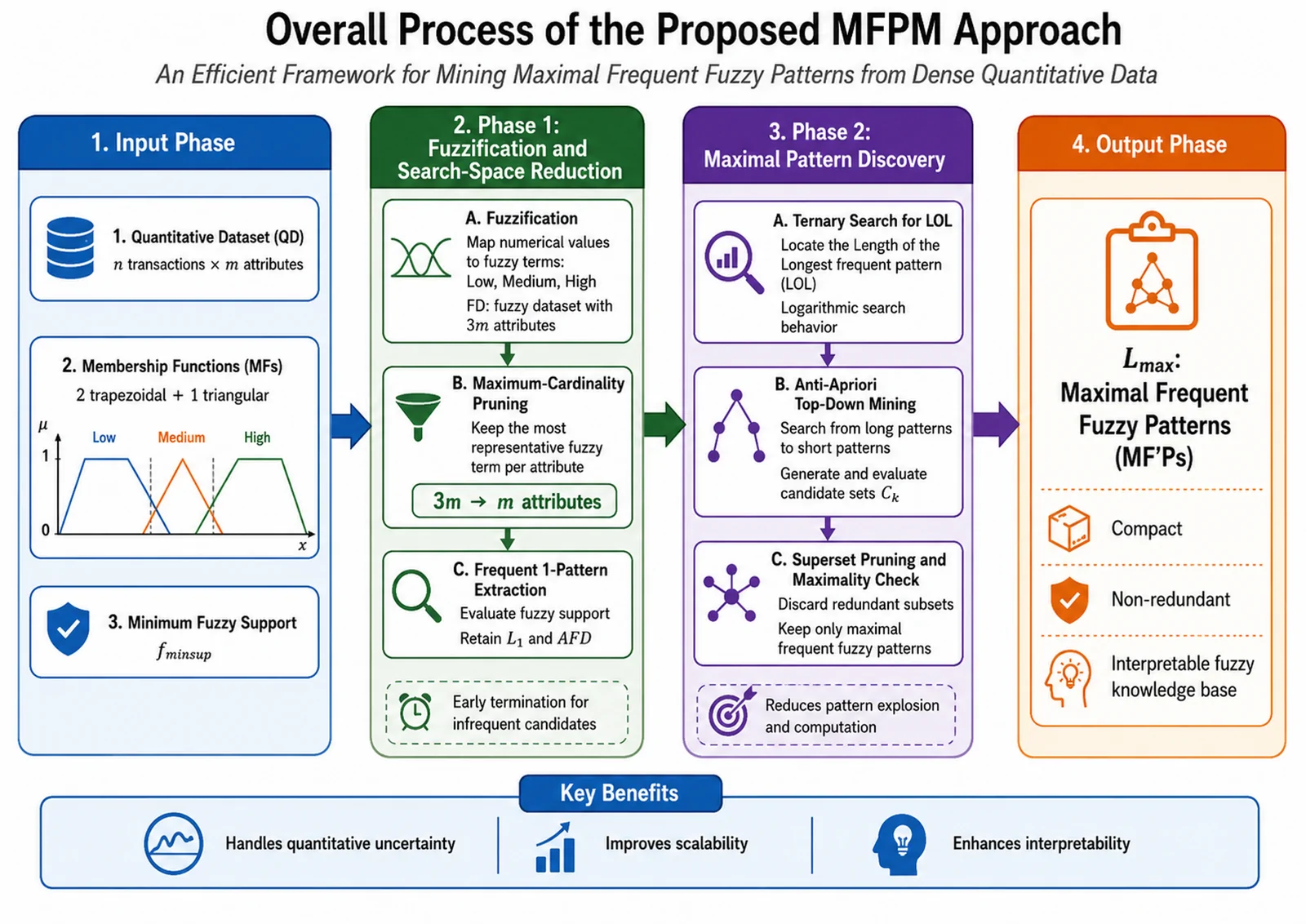
An Efficient Framework for Mining MF′Ps from Dense Quantitative Data.

The proposed MFPM methodology presents a structured and intelligent framework for transforming large quantitative datasets into compact, interpretable fuzzy knowledge as in Fig. 1. The Input Phase initializes the system through a quantitative dataset (QD), trapezoidal and triangular membership functions, and a fminsup, establishing the foundation for fuzzy modeling and support evaluation.

In Phase 1, the process integrates fuzzification and dimensionality reduction, converting numerical attributes into linguistic variables characterized by three fuzzy terms—Low, Medium, and High. Through cardinality pruning, only the most significant fuzzy term per attribute is preserved, effectively reducing data redundancy. The frequent 1-pattern filtering step identifies patterns meeting the *f*_*minsup*_ threshold, resulting in a compact fuzzy dataset that optimizes both accuracy and interpretability.

Phase 2 introduces an advanced discovery stage that embodies the novelty of this research. The ternary search mechanism efficiently locates the longest frequent pattern in logarithmic time, while the Anti-Apriori top-down strategy and superset pruning systematically eliminate redundant subsets, ensuring only maximal, non-overlapping fuzzy patterns are retained. This dual pruning mechanism significantly enhances computational efficiency and knowledge compactness.

Finally, the Output Phase yields a concise set of MF′Ps, forming a highly interpretable and semantically rich knowledge base. The proposed methodology demonstrates substantial improvements in scalability, pattern reduction, and interpretability, establishing it as a powerful framework for modern fuzzy data mining.

### Maximal frequent fuzzy pattern mining framework

The study presents the MFPM framework methodology, an innovative approach for identifying concise, consumable, and actionable patterns from complex quantitative datasets^35^. Traditional pattern mining algorithms suffer from interpretability issues and tend to generate hundreds or thousands of patterns, which are computationally inefficient for quantitative features. The MFPM framework aims to address these issues by developing a novel methodology that represents the primary contribution of this study^36^.

The novelty of the MFPM methodology lies in its synergistic integration of three distinct computational models, designed to work efficiently and effectively.

The methodological novelty of MFPM can be explained by four interrelated mechanisms. First, quantitative features are mapped to fuzzy linguistic terms, mitigating the sharp-boundary problem arising from fine-grained segmentation. Second, maximal-number pruning preserves the most representative fuzzy term for each feature, reducing the fuzzy search space from 3 m to 1 m while maintaining interpretability. Third, triple search is used to identify the longest recurring fuzzy pattern more efficiently than sequential exploration. Fourth, anti-Apriori downward mining, supported by superset pruning, directly extracts the most frequent fuzzy patterns and minimizes the evaluation of redundant sub-patterns. Consequently, MFPM provides a unified workflow that balances uncertainty modeling, scalability, and interpretability.

#### MFPM Algorithm: An integrated workflow

The MFPM approach provides a systematic, modular, and reproducible computational procedure for obtaining MF′Ps from quantitative datasets.

What distinguishes MFPM from other frequent pattern mining models is not that it has created a unique mining step, but instead that it orchestrates multiple mining steps non-redundantly, and is flexible, scalable, and interpretable. Specifically, MFPM integrates fuzzy membership functions into frequent pattern mining to overcome a key limitation of traditional approaches, namely the inability to represent uncertainty and vagueness in numerical data^37^.

The algorithm’s pipeline design ensures that each stage refines the dataset and feeds it into the next, enforcing scientific rigor and enabling seamless implementation across various application domains^38^.

quantitative Dataset: Let the dataset be defined as in Eq. 1.

1 $$\:QD=\left\{{T}_{1},{T}_{2},\dots\:,{T}_{n}\right\},{T}_{i}=\left({v}_{i1},{v}_{i2},\dots\:,{v}_{im}\right)\:\:\:\:$$

where n is the number of transactions and mmm the number of attributes, $\:i\in\:N$ is transaction index, $\:q\in\:N$ is attribute index, $\:{T}_{i}$ is the *i-th* transaction in the dataset, $\:{A}_{q}$ is the *q-th* quantitative attribute and $\:{v}_{iq}$is value of attribute $\:{A}_{q}$ in transaction $\:{T}_{i}$.

Fuzzification with Membership Functions: For each quantitative value $\:{v}_{im}$, its membership degree to a fuzzy linguistic term $\:F\in\:MFs$ is computed.

Example trapezoidal MF as in Eq. 2.

2 $$\:{\mu\:}_{\mathrm{trap}}\left(v;a,b,c,d\right)=\left\{\begin{array}{cc}0&\:v\le\:a\mathrm{\:or\:}v\ge\:d\\\:\frac{v-a}{b-a}&\:a<v\le\:b\\\:1&\:b<v\le\:c\\\:\frac{d-v}{d-c}&\:c<v<d\end{array}\right.$$

where $\:{\mu\:}_{trap}$ is trapezoidal membership function, v is input numerical value and * a,b,c,d* are breakpoint parameters defining the fuzzy region.

Example triangular MF as in Eq. 3.

3 $$\:{\mu\:}_{\mathrm{tri}}\left(v;a,b,c\right)=\mathrm{max}\left(0,\mathrm{m}\mathrm{i}\mathrm{n}\left(\frac{v-a}{b-a},\frac{c-v}{c-b}\right)\right)\:\:\:\:\:\:\:\:\:\:\:\:\:\:$$

where $\:{\mu\:}_{tri}$ is triangular membership function, *v* is input value.

The integration of dual trapezoidal and triangular membership functions enables nuanced modeling of real-world data uncertainty.

Fuzzy Support: For a fuzzy itemset $\:\stackrel{\sim}{I}$ as in Eq. 4.

4 $$\:\mathrm{Supp}\left(\stackrel{\sim}{I}\right)=\frac{1}{n}\sum\:_{i=1}^{n}{min}_{x\in\:\stackrel{\sim}{I}}{\mu\:}_{x}\left({T}_{i}\left[x\right]\right)\:\:\:\:\:$$

where *x* is fuzzy item in fuzzy itemset $\:\stackrel{\sim}{I}$, $\:{T}_{i}\left[x\right]$ is value of item *x* in transaction $\:{T}_{i}$ and $\:{\mu\:}_{x}(\cdot\:)$ is membership degree of fuzzy item *x.*

Inclusion criterion as in Eq. 5.

5 $$\:\stackrel{\sim}{I}\in\:L{\hspace{0.25em}\hspace{0.05em}}\Leftrightarrow{\hspace{0.25em}\hspace{0.05em}}\mathrm{Supp}\left(\stackrel{\sim}{I}\right)\ge\:{f}_{\mathrm{minsup}}\:\:\:\:\:\:\:$$

where *L* is set of frequent fuzzy itemsets and $\:{f}_{\mathrm{minsup}}$ is minimum fuzzy support threshold.

Extends classical frequency with fuzzy semantics, mitigating crisp threshold bias.

**Figure:**
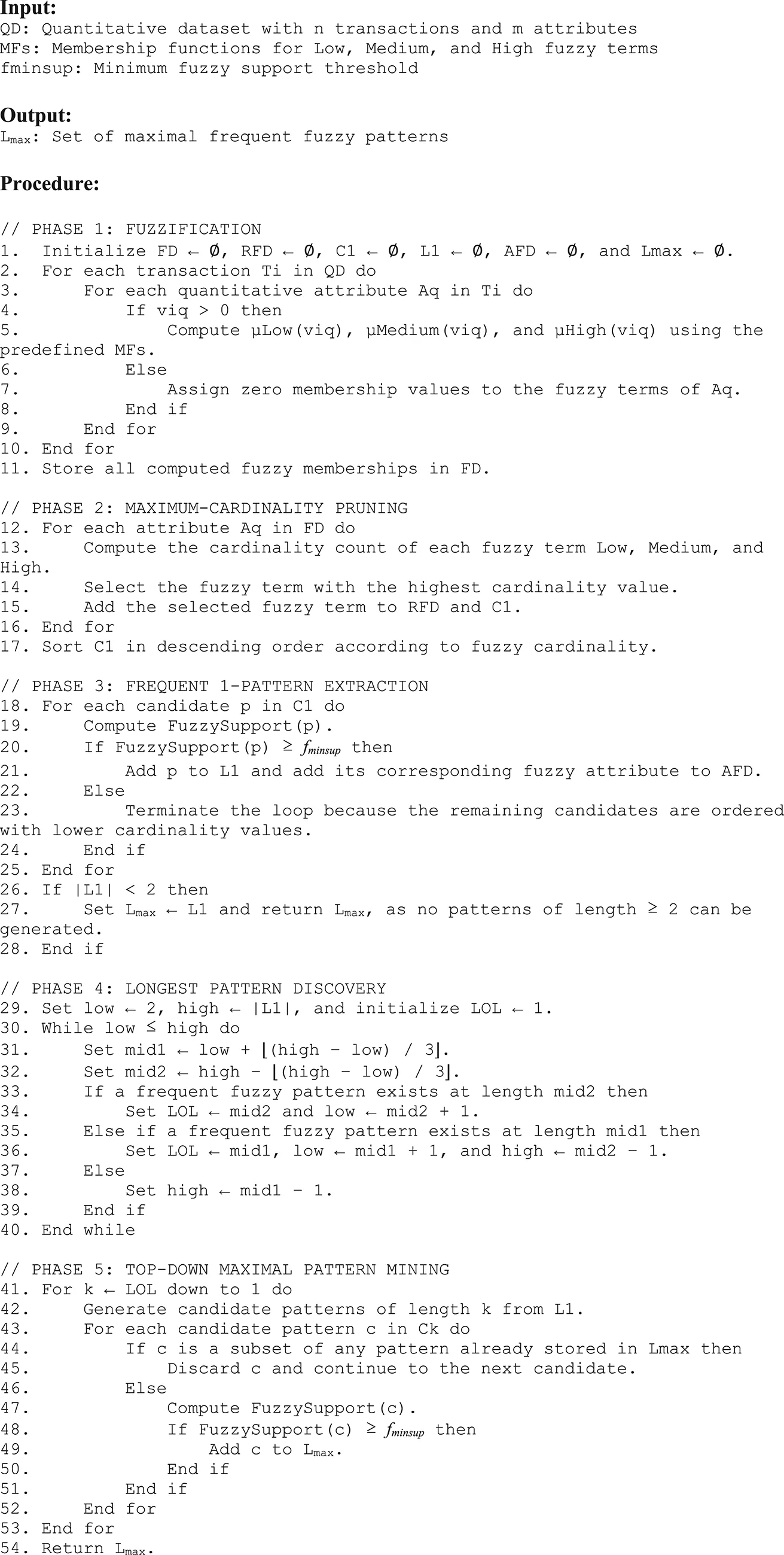
**Algorithm 1:** Maximal frequent fuzzy pattern mining MFPM.

Where low is lower bound of pattern length search, high is upper bound of pattern length search, 

$\:{mid}_{1},{mid}_{2}$ are ternary search midpoints, *k* is current pattern length and $\:{C}_{k}$: candidate patterns of length *k*.

**Table:** 

Main data structures used in algorithm 1	
**Symbol**	**Description**
FD	Full fuzzy dataset generated after fuzzification
RFD	Reduced fuzzy dataset after maximum-cardinality pruning
C1	Candidate 1-pattern set
L1	Frequent 1-pattern set
AFD	Active fuzzy dataset containing only frequent fuzzy terms
LOL	Length of the longest frequent fuzzy pattern
L_max_	Final set of maximal frequent fuzzy patterns

**Line-by-line explanation of algorithm** 1.

For clarity, the functional role of each major step in Algorithm 1 is explained below.

Line 1 initializes the main data structures used by the algorithm. FD stores the full fuzzy dataset, RFD stores the reduced fuzzy dataset, C1 stores candidate 1-patterns, L1 stores frequent 1-patterns, AFD stores the active fuzzy dataset, and L_max_ stores the final MF′Ps.

Lines 2–10 perform the fuzzification process. Each quantitative value is transformed into fuzzy membership degrees using the predefined membership functions in Eqs. (2) and (3). Positive values are mapped to Low, Medium, and High. Non-positive values are assigned zero membership values to avoid generating artificial fuzzy contributions for absent or inactive quantitative values.

Line 11 stores the complete fuzzy representation in FD. At this stage, each original quantitative attribute is represented by three fuzzy terms.

Lines 12–16 apply maximum-cardinality pruning. For each attribute, the algorithm computes the cardinality of its fuzzy terms using the cardinality measure in Eq. (6). It then keeps the fuzzy term with the highest cardinality value. This reduces the fuzzy search space from 3 m fuzzy terms to m selected fuzzy terms.

Line 17 sorts the candidate 1-patterns according to fuzzy cardinality. This ordering supports early termination in the next stage.

Lines 18–25 extract frequent 1-patterns. Each candidate is evaluated using fuzzy support, as defined in Eq. (4). Candidates satisfying *f*_*minsup*_ are added to L1 and retained in AFD. When the first infrequent candidate is reached, the loop terminates because C1 is sorted in descending cardinality order. For 1-patterns, this means the remaining candidates cannot satisfy the minimum fuzzy support threshold.

Lines 26–28 handle the case where fewer than two frequent 1-patterns exist. In this case, longer pattern generation is unnecessary, and the algorithm returns the available maximal result.

Lines 29–40 identify the length of the longest frequent fuzzy pattern. The ternary-search strategy evaluates two intermediate pattern lengths and iteratively narrows the search interval according to the existence of frequent fuzzy patterns.

Lines 41–53 perform top-down maximal frequent fuzzy pattern mining. The algorithm starts from the longest discovered length and moves toward shorter patterns. This Anti-Apriori strategy prioritizes longer candidate patterns before evaluating their subsets.

Line 42 generates candidate patterns of length k from L1.

Lines 43–46 apply superset pruning. If a candidate is already a subset of a previously discovered maximal frequent fuzzy pattern, it is discarded because it cannot be maximal.

Lines 47–50 evaluate the fuzzy support of the remaining candidates. If a candidate satisfies fminsup, it is added to L_max_ as a maximal frequent fuzzy pattern.

Line 54 returns L_max_, which represents the final set of non-redundant MF′Ps.

Each phase produces refined inputs for the next, yielding a transparent, reproducible workflow. Early pruning + LOL organization drastically reduce candidate explosion compared to Apriori-like methods. Fuzzification produces semantically meaningful patterns (e.g., High income & Medium expenditure). MFPM’s integration of trapezoidal and triangular membership functions, fuzzy support measures, and maximality checks yields compact, non-redundant pattern sets, addressing the “pattern explosion” problem in quantitative mining. Provides a generalizable, domain-independent methodology that balances efficiency, interpretability, and robustness, directly supporting applications in web science, remote sensing, and medical analytics^39^.

### Data transformation via fuzzification

The initial phase of the MFPM methodology addresses a fundamental limitation in quantitative data mining: the semantic gap between continuous numerical values and discrete pattern semantics. This phase implements a systematic transformation of the original quantitative dataset into a fuzzy linguistic representation, establishing the foundational layer for all subsequent pattern discovery operations.

A transformation process is applied to the quantitative dataset QD, which comprises n transactions of m attributes. For each quantitative value v_iq_ in the dataset, the algorithm maps the value to fuzzy sets utilizing specified membership functions. The algorithm progresses to each transaction Ti in QD, and each quantitative value v_iq_ in the transaction completes the conditional transformation. When the quantitative value v_iq_ is greater than 0, the algorithm computes a fuzzy set f_iq_ of membership degrees for each linguistic term, Low (µ_L_), Middle (µ_M_), and High (µ_H_), using the specified trapezoidal and triangular membership functions in MFs. For values < = 0, an empty fuzzy set f_∅_ is established to ensure strength in the transformations applied to varied dataset values.

Through this process, we end up converting every quantitative attribute to a linguistic variable, and the original m-dimensional dataset now transforms into a fuzzy dataset, FD, with 3 m attributes. For every original quantitative attribute, we have introduced three new attributes that represent the membership degrees of each value to the linguistic terms “Low,” “Middle,” and “High.” The fuzzy dataset retains the semantic richness of the original quantitative data while providing an appropriate representation for fuzzy pattern mining operations^40^.

The fuzzification process demonstrates the framework’s innovative integration of fuzzy set theory with pattern mining, capturing the uncertainty and graduality found in real quantitative data, while also preserving the mathematical rigor of the transformation process.

#### Formal foundation and implementation

In the fuzzification process, we implemented the mathematical framework introduced in Eqs. 2 and 3, which transformed each quantitative attribute R_q_ into a linguistic variable with terms {Low, Middle, High}. The algorithm calculates membership degrees for each value v_iq_ utilizing the MFs outlined below^41^:

Before membership-degree computation, each quantitative attribute is normalized into the interval [0, 1] to ensure that the membership-function parameters remain comparable across attributes with different numerical scales. For each attribute ($\:{A}_{q}$), strictly positive values are normalized using min–max normalization, as shown in Eq. 6:

6 $$\:\hat {{v}}_{iq}=\frac{{v}_{iq}-\mathrm{m}\mathrm{i}\mathrm{n}\left({A}_{q}^{+}\right)}{\mathrm{m}\mathrm{a}\mathrm{x}\left({A}_{q}^{+}\right)-\mathrm{m}\mathrm{i}\mathrm{n}\left({A}_{q}^{+}\right)}\:\:\:$$

where ($\:{A}_{q}^{+}$) denotes the set of positive observed values for attribute ($\:{A}_{q}$) and $\:\hat {{v}}_{iq}$ is normalized value in [0,1]. Non-positive values are not included in the normalization process and are assigned zero membership degrees for all fuzzy terms, consistent with Algorithm 1.

The normalized interval [0, 1] is divided into three overlapping linguistic terms: Low, Medium, and High. The Low and High terms are modeled using trapezoidal membership functions, while the Medium term is modeled using a triangular membership function. The exact membership-function parameter values are defined as follows:

Low = (0.00, 0.00, 0.25, 0.50), Medium = (0.25, 0.50, 0.75), and High = (0.50, 0.75, 1.00, 1.00). Accordingly, Low has full membership over [0.00, 0.25] and decreases gradually to zero at 0.50; Medium reaches maximum membership at 0.50 and decreases gradually toward 0.25 and 0.75; and High begins increasing at 0.50, reaches full membership at 0.75, and maintains full membership up to 1.00.

The use of three linguistic terms is motivated by interpretability and computational efficiency. The Low–Medium–High partition captures the main lower, intermediate, and higher tendencies of quantitative attributes while avoiding excessive fuzzy granularity. Since each additional linguistic term increases the dimensionality of the fuzzy dataset and enlarges the candidate search space, the three-term partition provides a practical compromise between semantic expressiveness, scalability, and output interpretability within the proposed MFPM framework.

- Trapezoidal MF (Eq. 2): Parameters (a, b, c, d) define the shoulders and core.
- Triangular MF (Eq. 3): Special case with b = c, creating symmetric membership.

The resulting fuzzy dataset *FD* expands the original m*m*-dimensional *QD* into 3*m* fuzzy attributes, where each original attribute *R*_*q*_ is represented by three new attributes $\:\left\{{\mu\:}_{L}\right({R}_{q}),{\mu\:}_{M}({R}_{q}),{\mu\:}_{H}({R}_{q}\left)\right\}$.

This conversion maintains the semantic depth of quantitative data while creating a more suitable representation for subsequent fuzzy pattern mining processes^42^.

The conditional treatment of non-positive values is further critical for improving various characteristics of a dataset and maintaining the mathematical consistency of the transformation itself. Furthermore, the just-described fuzzy dataset FD serves as the primary input for the subsequent candidate generation phase of the MFPM pipeline.

### Candidate 1-pattern generation with maximum cardinality pruning

This phase introduces the primary technique for addressing combinatorial problems in quantitative pattern mining^43^. The fuzzy dataset FD created from the fuzzification procedure contains 3 m fuzzy attributes, which can be drastically problematic for subsequent mining processes^44^. To help solve the problem, this process employs a maximum cardinality pruning strategy that maintains the truncation of the modeling itself, resulting in a significant reduction of the search space by retaining the most semantically valuable fuzzy representations^45^. This pruning strategy is based on fuzzy set theory and principles of information maximization. For each fuzzy term $\:{R}_{id}$ of attribute $\:{R}_{i}$ is calculated as Eq. 7^46^.

7 $$\:\mathrm{count}\left({R}_{id}\right)=\sum\:_{q=1}^{n}{\mu\:}_{{R}_{id}}\left({\mu\:}_{{R}_{id}}\left({v}_{iq}\right)\right)\:\:\:\:\:\:$$

where $\:{\mu\:}_{{R}_{id}}\left({v}_{iq}\right)$ is the membership degree of value $\:{\mu\:}_{{R}_{id}}\left({v}_{iq}\right)$ to fuzzy term $\:{R}_{id}$ and $\:d\in\:\{L,M,H\}$ is linguistic label index (Low, Medium, High).

This formulation represents the combined membership strength across all transactions and serves as a powerful measure of the importance of each term in the dataset^47^.

The originality of the algorithm lies in its heuristic selection criteria: given each original attribute, we only retain the fuzzy term with the maximum cardinality. This is mathematically justified according to the information-theoretic principle that the most representative term will carry the most discriminative power in constructing our patterns, as shown in Eq. 8.

8 $$\:{R}_{i,\mathrm{m}\mathrm{a}\mathrm{x}}=\mathrm{arg}\underset{d}{{max}\mathrm{count}\left({R}_{id}\right)}\text{}\:\:\:\:\:\:$$

where $\:{R}_{i,\mathrm{m}\mathrm{a}\mathrm{x}}$is preserved; all other fuzzy terms for R_i_ are pruned and $\:{R}_{i,\mathrm{m}\mathrm{a}\mathrm{x}}$ is selected dominant fuzzy term for attribute $\:{A}_{q}$.

ensures that each attribute contributes its most semantically meaningful representation to the candidate set.

The computational advantage is substantial: the dimensionality reduction from 3 m to m attributes represents a 66% decrease in search space complexity at this initial stage. This aggressive pruning strategy directly addresses the “curse of dimensionality” problem in fuzzy pattern mining, establishing a foundation for scalable processing of web-scale datasets.

Reduced Fuzzy Dataset (RFD): Original fuzzy dataset dimension: 3 m. After pruning became as in Eq. 9.

9 $$\:\mathrm{dim}\left(RFD\right)=m\:\:$$

Each attribute contributes one dominant fuzzy representation:

The sorted candidate set *C₁* facilitates efficient lattice traversal in subsequent phases, while the reduced fuzzy dataset *RFD* maintains the essential linguistic structure necessary for meaningful pattern discovery without the computational burden of redundant fuzzy representations. Unlike conventional Apriori-based approaches that retain all terms, MFPM introduces a heuristic-driven pruning step at the earliest stage, directly addressing the pattern explosion problem^48^.

#### Impact of maximum-cardinality pruning on pattern quality and completeness

The maximum-cardinality pruning strategy was adopted to reduce the high dimensionality of fuzzy pattern mining. Since each quantitative attribute $\:{A}_{q}$ is initially represented by three linguistic terms, namely Low, Medium, and High, the fuzzy representation expands the search space from *m* original attributes to 3 m fuzzy terms. Retaining only the fuzzy term with the highest aggregate membership strength therefore introduces a deliberate trade-off between exhaustive fuzzy representation and scalable maximal pattern discovery.

For each attribute $\:{A}_{q}$, let $\:{F}_{q,d}$ denote the fuzzy term associated with linguistic label $\:d\in\:\{L,M,H\}$. The selected fuzzy term is defined as Eq. 10:

10 $$\:{F}_{q,\mathrm{m}\mathrm{a}\mathrm{x}}=\mathrm{arg}\underset{d\in\:\left\{L,M,H\right\}}{{\:\:\:\:max}\:\:\:\:{count}\left({F}_{q,d}\right)}\:\:\:$$

Where $\:\mathrm{c}\mathrm{o}\mathrm{u}\mathrm{n}\mathrm{t}\left({F}_{q,d}\right)$ represents the aggregate membership contribution of the fuzzy term $\:{F}_{q,d}$ across all transactions. This selection preserves the dominant linguistic representation of each quantitative attribute and reduces redundant fuzzy alternatives before candidate generation.

Formally, let $\:{P}_{full}$ denote the complete fuzzy pattern space generated from all linguistic terms {Low, Medium, High} for all attributes, and let $\:{P}_{pruned}$ denote the reduced fuzzy pattern space obtained after retaining only the maximum-cardinality fuzzy term for each attribute. Since maximum-cardinality pruning removes some fuzzy terms before mining, the reduced pattern space is a subset of the full fuzzy pattern space as Eq. 11:

11 $$\:{P}_{pruned}\subseteq\:{P}_{full}\:\:\:\:\:$$

Accordingly, MFPM does not claim to provide a complete fuzzy representation that includes all linguistic terms. Instead, completeness is defined based on the reduced fuzzy representation resulting from maximal number trimming. This definition aligns with MFPM’s goal of extracting dominant, concise, non-repeating, and interpretable maximal fuzzy patterns, rather than enumerating all possible recurring fuzzy patterns.

From a pattern quality perspective, the retained fuzzy term is chosen because it makes the strongest cumulative contribution to membership across the dataset. However, this selection does not automatically qualify the term for inclusion in the final output. Each retained term must meet a fuzzy support threshold during the mono-recurring pattern extraction. Only candidates that meet this support threshold can participate in the subsequent top-down maximal mining process using the Apriori algorithm. Therefore, the final pattern quality is determined by fuzzy support, dominant membership stability, and maximal verification, not solely by the number of selected terms. However, the proposed pruning strategy is not loss-free. Fewer secondary fuzzy boundaries may be removed even if they contribute to rare, local, borderline, or class-specific patterns. Therefore, the pruning mechanism should be interpreted as an approximation strategy geared toward scalability and interpretability. It is best suited when the analytical goal is to identify the dominant maxima in dense quantitative datasets.

Future work could explore adaptive or uncertainty-accounting polynomial pruning strategies. For example, additional fuzzy boundaries could be retained when their number is close enough to the dominant boundary. Such expansions might achieve a better balance between coherence and improved pattern completeness in applications where minority or borderline fuzzy regions are analytically significant.

### Frequent 1-pattern extraction with early termination

This phase implements a critical filtering operation to identify genuinely frequent 1-patterns from the candidate set C₁. The methodology introduces a secondary pruning through an early termination mechanism that leverages the sorted structure of C₁ to achieve enhanced computational efficiency. The early termination strategy represents a significant optimization over conventional frequent pattern mining approaches^49^. The mathematical validity of this approach derives from the descending sort order of C₁ established during candidate generation. Since the candidate patterns are ordered by their cardinality values as in Eq. 12:

12 $$\:\begin{array}{cccc}&\:\mathrm{count}\left({\widehat{P}}_{1}\right){\hspace{0.25em}\hspace{0.05em}\hspace{0.25em}\hspace{0.05em}}\ge\:{\hspace{0.25em}\hspace{0.05em}\hspace{0.25em}\hspace{0.05em}count}\left({\widehat{P}}_{2}\right){\hspace{0.25em}\hspace{0.05em}\hspace{0.25em}\hspace{0.05em}}\ge\:{\hspace{0.25em}\hspace{0.05em}\hspace{0.25em}\hspace{0.05em}}\cdots\:{\hspace{0.25em}\hspace{0.05em}\hspace{0.25em}\hspace{0.05em}}\ge\:{\hspace{0.25em}\hspace{0.05em}\hspace{0.25em}\hspace{0.05em}count}\left({\widehat{P}}_{m}\right)&\:&\:\end{array}$$

The algorithm can safely terminate processing upon encountering the first infrequent candidate. This conclusion follows from the transitive property: if count $\:{\widehat{P}}_{k}$ < $\:{f}_{\mathrm{minsup}}$, then for all subsequent patterns where j > k, count $\:{\widehat{P}}_{j}$ as in Eq. 13.

13 $$\:\mathrm{count}\left({\widehat{P}}_{k}\right)<{f}_{\mathrm{minsup}}\Rightarrow\:\forall\:j>k,\mathrm{\:count}\left({\widehat{P}}_{j}\right)\le\:\mathrm{count}\left({\widehat{P}}_{k}\right)<{f}_{\mathrm{minsup}}\:\:\:\:\:$$

Where $\:\hat {{P}}_{j}$ is j-th candidate 1-pattern after sorting and j position index in ordered candidate set.

All subsequent candidates are also guaranteed to be infrequent.

The **frequency evaluation** applies the fuzzy support formulation introduced in Eq. (4), here specialized for 1-patterns as in Eq. 14.

14 $$\:\begin{array}{cccc}&\:\mathrm{Supp}\left(\widehat{P}\right)=\frac{\mathrm{count}\left(\widehat{P}\right)}{n}{\hspace{0.25em}\hspace{0.05em}\hspace{0.25em}\hspace{0.05em}}\ge\:{\hspace{0.25em}\hspace{0.05em}\hspace{0.25em}\hspace{0.05em}}{f}_{\mathrm{minsup}}&\:&\:\end{array}$$

This ensures full compatibility with the fuzzy mining framework, while retaining the **anti-monotonicity property** fundamental to efficient pattern discovery.

The procedure initializes an empty set for frequent 1-patterns L₁ and processes each candidate pattern P̂ in the sorted order of C₁. For each candidate, the algorithm evaluates whether its count value meets or exceeds the minimum fuzzy support threshold $\:{f}_{\mathrm{minsup}}$. Patterns satisfying this criterion are added to L₁, while the first pattern failing this condition triggers immediate termination of the evaluation loop^50^. A critical optimization check verifies whether the number of discovered frequent patterns |L₁| is less than two. This condition prevents futile attempts to discover longer patterns when insufficient frequent 1-patterns exist, thereby avoiding unnecessary computational overhead in sparse datasets. The final output consists of the frequent 1-pattern set L₁ and the actual fuzzy dataset AFD, which contains only the fuzzy attributes corresponding to patterns in L₁. This systematic approach to early termination within the MFPM process reveals a principled approach to increasing computational efficiency. In contrast to standard algorithms, which elaborate on all possible candidates by checking each one individually, MFPM provides a mathematical justification for a stop rule classification that exhibits order-independence, along with fuzzy support monotonicity, thereby establishing a robust and systematic foundation for scalable pattern discovery that is well-suited for large applications.

### Efficient longest pattern discovery via ternary search

In this section, a foundational and methodologically proposed is presented for determining the Length of the longest frequent fuzzy pattern (LOL). The traditional approach is to use a linear search and iterate from finding the LOL at |L₁| down to find the LOL at 2, thereby requiring O(n) complexity. The identified importance of the current methodology is that it can accomplish this efficiently, with a complexity of O (log₃ n ∕ log n), using a systematic searching process^51^. This ternary search approach method is an important improvement over longest pattern discovery method^52^. To start the algorithm, the search bounds are defined as follows: min = 2 and max = |L₁|. Instead of iteratively testing progressively longer patterns, the ternary search systematically tests three equal subranges of the overall search range [min, max] using two midpoints, which can be calculated as in Eqs. 15 and 16.

15 $$\:\begin{array}{cccc}&\:mi{d}_{1}=min+\lfloor\:\frac{(max-min)}{3}\rfloor\:&\:&\:\end{array}$$

16 $$\:\begin{array}{cccc}&\:mi{d}_{2}=max-\lfloor\:\frac{(max-min)}{3}\rfloor\:&\:&\:\end{array}$$

This partitioning allows the algorithm to discard one-third of the search space in each iteration, thereby achieving logarithmic complexity as in Eq. 17.

The computational contribution of ternary search should be interpreted at the pattern-length exploration level. Let $\:r=\mid\:{L}_{1}\mid\:$ denote the number of frequent 1-patterns after pruning and frequent 1-pattern filtering. A sequential longest-pattern discovery strategy may require testing up to * (r-1)* candidate lengths. In contrast, ternary search reduces the number of search iterations to $\:O\left({\mathrm{l}\mathrm{o}\mathrm{g}}_{3}r\right)$.

However, candidate generation and fuzzy support counting remain the dominant operations within each tested length. If $\:{C}_{k}$ denotes the candidate set generated at length (*k*), then the support-evaluation cost at that level can be expressed as $\:O(\mid\:{C}_{k}\mid\:\cdot\:n\cdot\:k)$, where (*n*) is the number of transactions and (k) is the candidate length. Thus, ternary search does not remove the intrinsic cost of candidate testing. Rather, it reduces the number of length levels for which candidate generation and support evaluation are triggered.

Accordingly, ternary search contributes to reducing the structural overhead of longest-pattern discovery, while the overall runtime behavior of MFPM results from the combined effect of maximum-cardinality pruning, early frequent 1-pattern filtering, ternary length exploration, and Anti-Apriori superset pruning.

17 $$\:\begin{array}{cccc}&\:T\left(N\right)=O\left({\mathrm{l}\mathrm{o}\mathrm{g}}_{3}N\right)&\:&\:\end{array}$$

The theoretical foundation derives from divide-and-conquer optimization principles, adapted specifically for fuzzy frequent pattern mining requirements.

For each iteration, candidate test combinations are generated from the frequent 1-pattern set L₁ at lengths mid₁ and mid₂ as in Eq. 18.

18 $$\:\begin{array}{cccc}&\:T{C}_{k}=\left(\genfrac{}{}{0pt}{}{\mid\:{L}_{1}\mid\:}{k}\right)&\:&\:\end{array}$$

For each candidate pattern $\:tc\in\:T{C}_{k}$ undergoes fuzzy support evaluation using the established measure as in Eq. 19.

19 $$\:\begin{array}{cccc}&\:Supp\left(tc\right)=\frac{1}{n}\sum\:_{i=1}^{n}\underset{x\in\:tc}{\mathrm{min}{\mu\:}_{x}\left({T}_{i}\left[x\right]\right)}{\hspace{0.25em}\hspace{0.05em}\hspace{0.25em}\hspace{0.05em}}\ge\:{\hspace{0.25em}\hspace{0.05em}\hspace{0.25em}\hspace{0.05em}}{f}_{\mathrm{minsup}}&\:&\:\end{array}$$

The search strategy adapts based on pattern frequency findings. If frequent patterns exist at mid₂, the search continues in the upper segment. If frequent patterns exist only at mid₁, the search proceeds in the middle segment. The absence of frequent patterns at both midpoints directs the search to the lower segment^53^. This novel method restricts discovery complexity from a linear, first-order pattern to a logarithmic, second-order pattern, effectively extracting large |L₁| values commonly found in web-scale, high-dimensional datasets. The function of divide-and-conquer optimization within a fuzzy frequent pattern mining context signifies a move away from tradition Apriori-style patterns. Still, it enables adaptive search capabilities while preserving the grounded computational nature. Additionally, the patterns remain semantically interpretable.

### Maximal pattern mining with anti-apriori and superset pruning

The last mining phase takes the core mining operation to extract the MF′Ps. This step involves a method that combines an Anti-Apriori style approach with a logic-based superset pruning aspect, so that maximal patterns are directly extracted when mining is completed, while removing any additional wasteful patterns of exploration or discovery^54^.

Anti-A Priori: In the mining phase of the algorithm, maximal pattern exploration occurs from the LOL in Phase Four, and then it will decrement the k maximal patterns in each successful iteration. This top-down search mode will ensure that if pattern c is discovered, all of its subsets will also not be maximal^55^. The strictly defined maximality criteria continue to hold, as described in Eq. 20.

20 $$\:\begin{array}{cccc}&\:c\in\:{L}_{\mathrm{m}\mathrm{a}\mathrm{x}}{\hspace{0.25em}\hspace{0.05em}\hspace{0.25em}\hspace{0.05em}}\Leftrightarrow{\hspace{0.25em}\hspace{0.05em}\hspace{0.25em}\hspace{0.05em}}Supp\left(c\right)\ge\:{f}_{\mathrm{minsup}}{\hspace{0.25em}\hspace{0.05em}}\wedge\:{\hspace{0.25em}\hspace{0.05em}}\nexists\:{c}^{{\prime\:}}\supset\:c:Supp\left({c}^{{\prime\:}}\right)\ge\:{f}_{\mathrm{minsup}}&\:&\:\end{array}$$

where $\:{c}^{{\prime\:}}$ proper superset of pattern c and $\:{L}_{\mathrm{m}\mathrm{a}\mathrm{x}}$ is maximal frequent fuzzy pattern set. This formal criterion guarantees that only patterns possessing no frequent supersets are retained within the final maximal pattern set $\:{L}_{\mathrm{m}\mathrm{a}\mathrm{x}}$.

Superset Pruning: A critical optimization mechanism involves the explicit elimination of candidate patterns that represent subsets of already discovered maximal patterns^56^. For each candidate $\:s\in\:{C}_{\mathrm{k}}$, check whether c is a subset of any pattern already discovered in $\:{L}_{\mathrm{m}\mathrm{a}\mathrm{x}}$. If such a superset exists, c is immediately pruned, as its maximality is violated as in Eq. 21.

21 $$\:s\in\:{L}_{\mathrm{m}\mathrm{a}\mathrm{x}}{\hspace{0.25em}\hspace{0.05em}\hspace{0.25em}\hspace{0.05em}such\:that\hspace{0.25em}\hspace{0.05em}\hspace{0.25em}\hspace{0.05em}}c\subset\:s\:\:\:\:\:\:\:$$

When this subset relationship holds, c is already discovered maximal pattern, preventing redundant computations. The fuzzy support calculation for composite patterns follows the established formulation in Eq. 22.

22 $$\:Supp\left(c\right)=\frac{count\left(c\right)}{n}\:\:\:\:\:\:$$

where *count(c)* represents the aggregated membership strength across all transactions.

The algorithm’s correctness is ensured through the anti-monotonicity principle, which states that if a candidate pattern is infrequent, all its supersets are necessarily infrequent. This property provides the theoretical foundation for safe pruning operations throughout the mining process^57^. The procedural implementation begins with initialization of the maximal pattern set $\:{L}_{\mathrm{m}\mathrm{a}\mathrm{x}}$ and sets the initial pattern length k to the predetermined LOL value. For each length k from LOL down to 2, the algorithm generates candidate combinations $\:{C}_{\mathrm{k}}$, from the frequent 1-pattern set L₁. Each candidate undergoes superset checking against already discovered maximal patterns in $\:{L}_{\mathrm{m}\mathrm{a}\mathrm{x}}$, with redundant candidates being eliminated. The remaining candidates are evaluated for frequency compliance, and those meeting the support threshold are added to $\:{L}_{\mathrm{m}\mathrm{a}\mathrm{x}}$^58^.

This methodology represents a significant departure from classical bottom-up Apriori-style approaches. Through the integration of Anti-Apriori search, superset dominance pruning, and fuzzy support monotonicity within a unified framework, MFPM generates a compact, non-redundant set of MF′Ps, ensuring both computational efficiency and semantic interpretability for practical applications^59^.

## Results and evaluation

### Dataset preparation and fuzzification

This section provides a detailed assessment of the proposed MFPM framework in terms of its effectiveness and efficiency compared to benchmarks. Specifically, the experimental assessment utilizes three dense benchmark datasets from the FIMI repository: (a) Chess; (b) Connect; and (c) Mushroom, characteristics of which are reported in Table 1. Binary datasets were modified to be quantitative datasets by substituting present attributes with integers (1–100) assigned randomly and absent attributes with zero, thereby establishing more challenging conditions for the fuzzy pattern discovery task.

As the MFPM framework evaluation seeks to address a slightly larger issue in quantitative pattern mining: the absence of large-scale datasets with native fuzzy or quantitative attributes. Although the methodology focuses on quantitative data extraction, a methodological approach is warranted for method validation, utilizing established dense binary datasets from the Frequent Itemset Mining Dataset Repository (FIMI). During the validation process, the proposed MFPM framework follows conventional practice in pattern mining, where binary datasets are systematically modified into more challenging quantitative environments.

The systematic methodology used in the conversion process follows a valid approach whereby absent binary attributes are assigned a value of zero, and present binary attributes receive a value assigned, uniformly distributed between 1 and 100 (i.e., a random integer is assigned). Through this conversion, both fully dense quantitative datasets are generated, containing positive quantities (i.e., number values between 1 and 100) along with the previously assigned zero value, which represents the absent binary attributes and is now reflected as a zero. However, generating fully dense quantitative datasets creates a more challenging evaluation environment for assessing the robustness of the framework in demanding contextual conditions.

In the fuzzification step, the framework utilizes well-documented membership functions from the area of fuzzy set theory. To this end, two trapezoidal functions and a triangular function convert each quantitative value into a fuzzy set that includes three associated linguistic terms—Low, Medium, and High. The data sets chosen for this study—Chess, Connect, and Mushroom—exhibit a variety of transaction volumes, attribute dimensions, and pattern densities, allowing for thorough testing across different application scenarios. This methodological strategy will reinforce that the performance metrics clearly illustrate the framework’s ability to meet the complexities and dimensionality constraints of real-world applications in web science.

**Table 1:** Characteristics of datasets used in the experiments.

No.	Dataset name	Number of transactions	Number of attributes	Average transaction length	Dataset type
1	Chess	3,196	75	35	Dense
2	Connect	67,557	129	43	Dense
3	Mushroom	8,124	119	23	Dense

### Evaluate the proposed system on chess dataset

The Chess dataset, comprising 3,196 transactions and 75 binary attributes with an average transaction length of 35, is the first dataset used to evaluate the MFPM framework. Given the quantitative aspect of the proposed approach, a methodical transformation was applied in which absent attributes were coded as zero, and present attributes had an integer value randomly assigned between 1 and 100. The transformation produced a dense quantitative dataset that mimics real-world quantitative data environments.

The fuzzification process employed predefined membership functions to convert quantitative values into fuzzy representations. Table 2 illustrates the resulting fuzzy dataset for the first five attributes across three sample transactions. Each quantitative value is transformed into a fuzzy set characterized by membership degrees for three linguistic terms: Low (L), Medium (M), and High (H). For instance, the quantitative value 73 of attribute A1 in transaction 1 demonstrates partial membership in both Medium (0.23) and High (0.77) categories, effectively capturing the uncertainty inherent in quantitative data through graded membership assignments.

**Table 2:** Sample transactions from fuzzy chess dataset.

TID	A1			A2			A3			A4			A5		
	L	M	H	L	M	H	L	M	H	L	M	H	L	M	H
1	0	0.23	0.77	0	0	0	0	0.44	0.56	0	0	0	0	0.13	0.87
2	0.83	0.17	0	0	0	0	0.13	0.87	0	0	0	0	0	0.3	0.7
3	0	0	1	0	0	0	0	1	0	0	0	0	0	0	1

#### Maximal Frequent Fuzzy Pattern Discovery

The MFPM framework was applied to the fuzzy Chess dataset with varying minimum support thresholds (f_minsup_) to evaluate its pattern discovery capabilities. Table 3 presents the number of MF′Ps identified at different support levels, demonstrating the framework’s ability to produce compact pattern sets while maintaining comprehensive coverage.

**Table 3:** MFPs Discovered from Chess Dataset.

f_minsup_	Number of MF′Ps
0.2	369
5	653
50	128
100	70
200	13
250	4

The findings reveal multiple relevant aspects of the proposed methodology. The framework reported approximately 369 MFPs at the minimum support threshold (*f*_*minsup*_ = 0.2) and therefore provided extensive coverage of all patterns. As the support threshold increased, the number of patterns uncovered was drastically reduced — e.g., exactly four patterns remained at *f*_*minsup*_ = 250: (A21.H, A25.H), (A15.H, A17.L), (A25.H, A17.L), and (A17.L, A19.L). It is notable that the discovered patterns have the highest support thresholds, indicating that these patterns demonstrate the highest meaningful attribute relationships in the dataset. The fuzzy counts for A21 and A25 are 262.0, A15 and A17 are 274.0, A25 and A17 are 295.4, and A17 and A19 are 274.7.

The patterns discovered indicate that the framework can identify meaningful linguistic associations, such as co-occurrences of high values of attributes A21 and A25, or A15 having high values when A17 has low values. This ability to identify both linguistically rich and nuanced understanding of co-occurring attributes while retaining compactness via maximal pattern mining provides a solution for both usability and scalability in quantitative data mining.

The rate at which the patterns decreased with support thresholds helped validate that the algorithms filtered redundant patterns while maximizing length-one, length-two, and length-three patterns. This is especially meaningful for the stakeholders are interested in deriving actionable inference without inundating them with a pattern explosion, an issue with traditional frequent pattern mining methods.

### Evaluate the proposed system on high-dimensional mushroom dataset

The Mushroom dataset serves as an extensive benchmark for evaluating pattern mining algorithms, consisting of 8,124 instances represented by 119 unique binary features. With an average of 23 features per instance, the dataset presents significant challenges to pattern discovery algorithms and thus requires a high dimensionality. Table 4 showcases the first three instances, demonstrating the combinatorial complexity of the dataset and its patterns in feature distribution.

**Table 4:** Initial Three Instances of the Mushroom Dataset.

TID	Features
1	F1, F3, F9, F13, F23, F25, F34, F36, F38, F40, F52, F54, F59, F63, F67, F76, F85, F86, F90, F93, F98, F107, F113
2	F2, F3, F9, F14, F23, F26, F34, F36, F39, F40, F52, F55, F59, F63, F67, F76, F85, F86, F90, F93, F99, F108, F114
3	F2, F4, F9, F15, F23, F27, F34, F36, F39, F41, F52, F55, F59, F63, F67, F76, F85, F86, F90, F93, F99, F108, F115

Following the established experimental methodology, binary features were systematically transformed into quantitative representations. The present features received randomly assigned integer values between 1 and 100, while zeros denoted the absence of features. This transformation generated a dense quantitative dataset, which was subsequently converted into a fuzzy representation using predefined membership functions, enabling the application of the MFPM framework.

#### Maximal pattern extraction and analysis

The application of the MFPM framework to the fuzzy Mushroom dataset yielded significant results in terms of pattern compactness and quality. Table 5 presents the number of MF′Ps discovered at varying support thresholds, demonstrating the framework’s effectiveness in high-dimensional environments.

**Table 5:** MFPs Discovered from Mushroom Dataset.

f_minsup_	Number of MFPs
0.5	28
10	27
125	9
250	2

The results reveal remarkable pattern compression capabilities. Even at minimal support thresholds (f_minsup_= 0.5), the framework identified only 28 maximal patterns, representing an exceptional dimensionality reduction from the original 119 features. This compression becomes increasingly pronounced at higher support levels, with only two patterns remaining at f_minsup_= 250: (F1.H, F110.H) with a fuzzy count of 406.165 and (F10.H, F110.H) with a count of 252.861.

The discovered patterns demonstrate significant semantic value, revealing strong associations between specific feature combinations. The consistent presence of feature F110.H in both high-support patterns indicate its central role in the dataset’s structure, while the co-occurrence with either F1.H or F10.H suggests distinct feature relationship patterns. These findings align with established knowledge in pattern mining literature regarding core feature identification in high-dimensional datasets.

The stability in pattern counts between f_minsup_= 0.5 (28 patterns) and f_minsup_= 10 (27 patterns) indicates the presence of robust feature associations that persist across support thresholds. This characteristic is particularly valuable for practical applications where pattern stability across parameter variations is essential for reliable knowledge discovery.

The extreme pattern compression achieved—from 119 potential feature combinations to only two maximal patterns at high support—demonstrates the framework’s capability to extract the most significant relationships while eliminating redundancy. This addresses a fundamental challenge in high-dimensional data mining, providing domain experts with actionable insights without overwhelming them with pattern proliferation.

### Analysis of high-utility fuzzy pattern mining on the connect dataset

The performance of the proposed high-utility fuzzy pattern mining algorithm was evaluated on the Connect dataset, a dense dataset comprising 67,557 transactions across 129 binary items. The initial records, illustrated in Table 6, demonstrate the dataset’s high density, with each transaction containing a substantial number of the available items. To facilitate fuzzy utility mining, these binary transactions were first converted into a quantitative format, as shown in Table 7, where present items were assigned random positive values. This quantitative data was subsequently fuzzified into Low (L), Medium (M), and High (H) linguistic terms using predefined membership functions, a sample of which is presented in Table 8.

**Table 6:** Sample Records from the Original Connect Dataset.

TID	Items (Subset Shown for Brevity)
1	I1, I4, I7, I10, I13, I16, I19, I22, I25, I28, I31, I34, I37, I40, I43, I46, I49, I52, I55, I58, I61, I64, I67, I70, I73, I76, I79, I82, I85, I88, I91, I94, I97, I100, I103, I106, I109, I112, I115, I118, I121, I124, I127
2	I1, I4, I7, I10, I13, I16, I19, I22, I25, I28, I31, I34, I37, I40, I44, I46, I49, I52, I55, I58, I61, I64, I67, I70, I73, I77, I79, I82, I85, I88, I91, I94, I97, I100, I103, I106, I109, I112, I115, I118, I121, I124, I127
3	I1, I4, I7, I10, I13, I16, I19, I23, I25, I28, I31, I34, I37, I40, I44, I46, I49, I52, I55, I58, I61, I64, I67, I70, I73, I76, I79, I82, I85, I88, I91, I94, I97, I100, I103, I106, I109, I112, I115, I118, I121, I124, I127

**Table 7:** Sample of the Generated Quantitative Connect Dataset.

TID	I1	I2	I3	I4	…	I127	I128	I129
1	25	0	0	48	…	18	0	0
2	88	0	0	56	…	29	0	0
3	9	0	0	71	…	78	0	0

**Table 8:** Sample of the Fuzzy Connect Dataset.

TID	I1			12			13				1129		
	L	M	H	L	M	H	L	M	H	…	L	M	H
1	0	0.24	0.76	0	0	0	0	0.93	0.07	…	0	0	0
2	0	0	1	0	0	0	0	0.8	0.2	…	0	0	0
3	1	0	0	0	0	0	0	0.3	0.7	…	0	0	0

A key parameter in the mining process is the minimum fuzzy support (f_minsup_), which controls the minimum count required for a pattern to be considered frequent. The algorithm’s sensitivity to this threshold is clearly demonstrated in Table 9. At a very low *f*_*minsup*_ of 0.5, a large number of patterns (48) are discovered. Notably, the count of discovered Most Interesting Fuzzy Patterns (MF´Ps) initially increased to 62 as *f*_*minsup*_ was raised to 5, suggesting the algorithm first stabilizes by filtering out noise before progressively eliminating less frequent patterns at higher thresholds. A stringent *f*_*minsup*_ of 3000 was selected to isolate only the most prevalent and significant patterns, resulting in a concise set of 13 MF´Ps.

**Table 9:** Number of MF´Ps Discovered at Various Minimum Fuzzy Support (fminsup) Thresholds.

f_minsup_	Number of MF´Ps Discovered
0.5	48
1	54
5	62
100	36
1000	15
2000	17
**3000**	**13**

The final output at *f*_*minsup*_ = 3000, detailed in Table 10, reveals the core high-utility associations within the dataset. The most critical finding is the exclusive dominance of a specific item set {I1, I10, I100, I103, I106, I109} and the exclusive presence of the “High” (H) fuzzy linguistic state across all 13 MF´Ps. This indicates that the most significant patterns in the Connect dataset are driven by the co-occurrence of these particular items with high quantitative values. The structure of the output, which includes both 2-item and 3-item sets, efficiently conveys non-redundant information. For instance, the 3-item pattern (I103.H, I109.H, I106.H) with a count of 3466.5 subsumes its constituent 2-item pairs; by reporting only the MF´Ps, the algorithm provides a compact summary without the redundancy of all smaller, frequent subsets. This enhances the interpretability of the results, directing attention to the most complex and utility-rich associations.

**Table 10:** Most Interesting Fuzzy Patterns (MF´Ps) Discovered at fminsup = 3000.

No.	Most Interesting Fuzzy Pattern (MF´*P*)	Count Value
1	(I100.H, I103.H, I109.H)	3160.4
2	(I100.H, I103.H, I106.H)	3212.4
3	(I100.H, I109.H, I106.H)	3151.8
4	(I103.H, I109.H, I106.H)	3466.5
5	(I100.H, I1.H)	5210.1
6	(I100.H, I10.H)	6461.4
7	(I103.H, I1.H)	5694.8
8	(I103.H, I10.H)	7040.6
9	(I109.H, I1.H)	5932.1
10	(I109.H, I10.H)	7336.8
11	(I106.H, I1.H)	5972.9
12	(I106.H, I10.H)	7334.4
13	(I1.H, I10.H)	4903.4

### Performance evaluation of effectiveness in pattern reduction

This section presents a comprehensive evaluation of the proposed MFPM framework against established benchmarks in frequent pattern mining. The discussion synthesizes empirical findings from multiple datasets to assess the framework’s novelty, effectiveness, and practical utility while contextualizing its performance within the broader research landscape of quantitative data mining.

The effectiveness of the MFPM framework is most evident in its ability to produce compact, non-redundant pattern sets while preserving essential knowledge. Comparative analysis against the benchmark FTDA algorithm across three dense datasets reveals remarkable pattern reduction capabilities. The data presented in Table 11 demonstrates a consistent and substantial pattern reduction across all datasets and support thresholds. Notably, the performance on the Chess dataset at *f*_*minsup*_ = 0.2 is particularly noteworthy, where MFPM achieved a 94.97% reduction in output patterns (from 7,334 to 369) while maintaining an equivalent knowledge representation. This reduction is not merely quantitative but qualitative—by eliminating redundant sub-patterns, MFPM directs analytical attention to the most informative relationships within the data.

**Table 11:** Comparative Pattern Discovery: MFPM vs. FTDA.

Dataset	f_minsup_	FTDA Patterns	MFPM Patterns	Reduction Rate
Chess	0.2	7,334	369	94.97%
Chess	5	1,473	653	55.67%
Chess	250	21	4	80.95%
Connect	0.5	454	48	89.43%
Connect	5	309	62	79.94%
Connect	3,000	25	13	48.00%
Mushroom	0.5	114	28	75.44%
Mushroom	10	69	27	60.87%
Mushroom	250	11	2	81.82%

The pattern compression effect becomes increasingly valuable in real-world applications that require human analysis. Traditional approaches, such as FTDA, generate pattern explosions that overwhelm domain experts, whereas MFPM produces manageable sets of maximal patterns from which all sub-patterns can be derived. This addresses a critical practical limitation in previous F´PM implementations and enhances the framework’s applicability to knowledge discovery tasks in domains such as healthcare, market basket analysis, and web usage mining.

### Performance evaluation of efficiency in computational time

Beyond pattern reduction, the MFPM framework demonstrates significant improvements in computational efficiency, particularly under challenging conditions of low support thresholds and dense datasets. The experimental results reveal a consistent performance advantage for MFPM compared to the FTDA benchmark. The runtime comparisons in Table 12 highlight MFPM’s superior efficiency, particularly at lower *f*_*minsup*_ thresholds where computational complexity is highest. On the Chess dataset at *f*_*minsup*_ = 0.2, MFPM completed the mining process in 572 s compared to FTDA’s 1,653 s—a 65.42% improvement. This performance advantage stems from MFPM’s innovative search strategy and pruning techniques.

**Table 12:** Comparative Runtime Performance: MFPM vs. FTDA (seconds).

Dataset	f_minsup_	FTDA Runtime	MFPM Runtime	Speed Improvement
Chess	0.2	1,653	572	65.42%
Chess	5	174	66	62.07%
Connect	0.5	1,369	736	46.24%
Connect	5	1,423	759	46.66%
Connect	3,000	4	3	25.00%
Mushroom	0.5	126	41	67.46%
Mushroom	125	6	3	50.00%

The runtime improvements reported in Table 12 reflect the combined effect of the proposed MFPM components rather than the isolated effect of ternary search alone. Candidate generation and fuzzy support counting remain the dominant computational operations. Within this context, ternary search contributes by reducing the number of pattern-length levels evaluated during longest frequent pattern discovery. Therefore, the observed runtime improvement should be interpreted as the joint result of maximum-cardinality pruning, early frequent 1-pattern filtering, ternary length exploration, and Anti-Apriori superset pruning.

Unlike FTDA’s bottom-up, level-wise approach that must generate and test all possible pattern combinations, MFPM employs a long-to-short (Anti-Apriori) search strategy. This approach begins by identifying the longest possible frequent patterns first, then progressively explores shorter patterns. More importantly, MFPM incorporates two novel pruning strategies:

Superset Pruning: Candidate patterns that are subsets of already discovered maximal patterns are eliminated without count computation.

Subset Infrequent Pruning: During count calculation, patterns identified as infrequent through preliminary fuzzy intersections are pruned early.

These strategies collectively reduce the search space exponentially, providing the computational advantages observed in the experimental results. The performance gap is most pronounced at lower support thresholds because these conditions create more potential pattern combinations, magnifying the benefits of MFPM’s pruning mechanisms.

### Controlled mining-phase scalability analysis with respect to transaction volume and dimensionality

A scalable, controlled experiment was conducted to evaluate the behavior of the MFPM algorithm during the data extraction phase as transaction size and dimensionality increased. Connect was chosen as the largest metric, comprising 67,557 transactions and 129 attributes. The stored quantum dataset from the original experiment was reused unchanged. Preprocessing, affiliation function calibration, program settings, and subset construction were maintained.

The size and dimensions of the transactions were changed independently. Nested transaction subsets contained 16,889, 33,779, 50,668, and 67,557 transactions, retaining all 129 attributes. Nested dimension subsets contained 32, 65, 97, and 129 attributes, retaining all 67,557 transactions.

The overlapping sub-dimension sets contained 32, 65, 97, and 129 features, retaining all 67,557 coefficients. Relative fuzzy support was defined as follows Eq. 23:

23 $$\:RF{S}_{s}\left(X\right)=\frac{F{C}_{s}\left(X\right)}{{n}_{s}}\:\:\:\:\:\:\:\:$$

The threshold was fixed, $\:\tau\:=\frac{100}{\mathrm{67,557}}\approx\:0.0014802315$ giving fuzzy-count thresholds of $\:\tau\:\cdot\:{n}_{s}\in\:\left\{24.99962994,{\hspace{0.25em}\hspace{0.05em}}50.00074012,{\hspace{0.25em}\hspace{0.05em}}75.00037006,{\hspace{0.25em}\hspace{0.05em}}100.00000000\right\}$ for the transaction subsets. Real-valued thresholds were retained. All dimensional configurations used: $\:{f}_{minsup}=100$.

Each configuration included an unmeasured preliminary round, followed by five measured rounds in separate operations. Timing began after data loading, quantization, and fuzzing, and ended upon the return of the most frequent fuzzy pattern. Therefore, the reported values represent only the runtime of the mining phase. RSS peaks were sampled every 10 ms using psutil and are reported as mean ± standard deviation. Support counts were calculated whenever fuzzy support was evaluated during recurring single-pattern extraction, detection of the longest pattern based on triple search, or maximal top-down mining. Because the procedure was deterministic under constant data and settings, these numbers are reported as integers.

With transaction size increasing nearly fourfold, runtime increased from 28.4 ± 1.2 s to 433.6 ± 8.7 s, a 15.3-fold increase (see Table 13). Peak RSS data size rose from 847 ± 32 MB to 1562 ± 58 MB. Backup account calls increased by 35.6%, from 12,847 to 17,423, while the total $\:\mid\:{L}_{1}\mid\:$ search data size increased from 87 to 96. The number of peak recurring fuzzy patterns was 12, 14, 13, and 36, respectively. This large non-linear growth in runtime may reflect the combined effects of evaluating retained patterns on larger transaction subsets, increased memory and data access costs, and changes in the active search space.

**Table 13:** Transaction-Volume Scalability of MFPM.

Data fraction	Transactions	Attributes	|L₁|	|L_max_|	Runtime (s), mean ± SD	Peak RSS (MB), mean ± SD	Support-computation calls
25%	16,889	129	87	12	28.4 ± 1.2	847 ± 32	12,847
50%	33,779	129	91	14	98.7 ± 3.4	1,124 ± 41	14,891
75%	50,668	129	94	13	221.3 ± 6.1	1,389 ± 52	16,234
100%	67,557	129	96	36	433.6 ± 8.7	1,562 ± 58	17,423

Increasing the number of dimensions from 32 to 129 attributes increased runtime from 67.3 ± 2.1 s to 433.6 ± 8.7 s, a 6.44-fold increase (see Table 14). The amount of data used for support calculation (RSS) also increased by a factor of 2.51, from 623 ± 28 MB to 1562 ± 58 MB. Support calculation calls increased by a factor of 2.12, from 8234 to 17,423, while the total $\:\mid\:{L}_{1}\mid\:$ increased by a factor of 2.82, from 34 to 96. Maximum recurrent fuzzy patterns increased from 6 to 36. The slower increase in support calculation calls compared to the increase in the number of dimensions suggests that the pruning process limited the growth of support evaluation, although the total runtime also depends on the filter length, data access, and the cost of evaluation per call.

The full configuration was performed once and reused in both analyses. It reproduced 36 recurrent fuzzy patterns at a maximum of $\:{f}_{minsup}=100$, consistent with the original Connect result. The same full configuration was used as the common endpoint for both experiments. Its runtime was not used as a proxy for previous comprehensive timing results because the current analysis excludes preprocessing and fuzzy optimization. The analysis is limited to a single device, a single constant attribute switch, and mining-stage measurements. Multiple switches, comprehensive measurements, and larger datasets remain among the directions to be explored in future work.

**Table 14:** Dimensional Scalability of MFPM.

Attribute fraction	Transactions	Attributes	|L₁|	|L_max_|	Runtime (s), mean ± SD	Peak RSS (MB), mean ± SD	Support-computation calls
25%	67,557	32	34	6	67.3 ± 2.1	623 ± 28	8,234
50%	67,557	65	58	18	148.7 ± 4.2	978 ± 36	11,567
75%	67,557	97	78	27	276.4 ± 6.8	1,287 ± 47	14,892
100%	67,557	129	96	36	433.6 ± 8.7	1,562 ± 58	17,423

## Discussion and comparative analysis

This study demonstrates that the MFPM framework successfully addresses the critical challenge of pattern explosion in quantitative data mining. By integrating maximal pattern representation with fuzzy set theory, it achieves substantial pattern reduction and computational efficiency while maintaining comprehensive knowledge representation. The principal limitation in existing fuzzy pattern mining literature concerns the computational intractability and interpretative challenges arising from pattern explosion. While numerous studies have advanced fuzzy mining techniques, they predominantly focus on extracting complete sets of fuzzy frequent patterns. This approach becomes computationally prohibitive in dense quantitative datasets, where the combinatorial explosion of potential patterns overwhelms both processing capabilities and human analytical capacity. Previous methodologies, including those employing advanced data structures like fuzzy FP-Trees, fail to adequately address the fundamental redundancy inherent in pattern enumeration, where numerous discovered patterns represent trivial subsets of more comprehensive relationships.

To broaden the scope of the comparison to include areas beyond FTDA, the proposed MFPM framework was compared with the latest cutting-edge methods in relevant pattern extraction categories, as summarized in Table 15. These methods include Apriori-based fuzzy pattern extraction, maximal pattern growth methods, closed and closed fuzzy pattern extraction, fuzzy pattern growth methods, type II fuzzy pattern extraction, top-k element extraction, distributed and rule-based extraction, and graph-based maximal extraction. These methods offer significant advancements in scalability, redundancy reduction, fuzzy uncertainty modeling, time-based pattern extraction, graph-based pattern extraction, and rule-guided knowledge discovery. However, they generally focus on improving a single, specific dimension of the extraction problem, rather than addressing fuzzy quantum representation, maximal pattern coherence, and pattern explosion in dense data within a single framework.

FTDA was retained as the primary quantitative criterion because it is the closest direct baseline for evaluating fuzzy recurring pattern detection using convergent measures of pattern count and runtime. In contrast, many modern approaches produce different types of outputs, such as closed element sets, higher-frequency element sets (higher than k), time patterns, binary sets, distributed correlation rules, or profit-supported fuzzy rules. Therefore, a direct comparison of runtime or pattern count with these methods may be methodologically unfair without modifying their objectives or output definitions.

Accordingly, the revised research distinguishes between a direct empirical comparison and a broader systematic comparison. The direct empirical comparison is performed using FTDA, while Table 15 presents a systematic comparison with additional representative approaches whose objectives, data models, or output semantics differ from MFPM. A direct comparison of runtime with Apriori fuzzy algorithms or FP-based fuzzy growth algorithms was not performed because these methods typically produce all recurring fuzzy patterns rather than maximal recurring fuzzy patterns. Directly comparing its runtime with MFPM requires either adjusting the output targets or applying post-processing to extreme patterns, which can introduce confounding factors affecting the fairness of the comparison. As shown in Table 12, MFPM achieves significant runtime improvements compared to FTDA under directly comparable experimental conditions, including a 65.42% improvement on the Chessboard dataset at (*f*_*minsup*_ = 0.2). This supports the effectiveness of the proposed pruning and searching mechanisms in a similar environment for extracting fuzzy recurring patterns.

**Table 15:** Methodological Positioning of MFPM against Representative State-of-the-Art Pattern Mining Approaches.

Category	Representative approaches	Main focus	Difference from MFPM
Apriori-based fuzzy mining	FTDA	Fuzzy frequent pattern mining with candidate generation	Generates broad fuzzy frequent pattern sets and may suffer from candidate explosion in dense datasets
Maximal pattern-growth	FT-FP-tree^16^	Maximal or fault-tolerant frequent itemsets	Does not incorporate fuzzy membership functions for quantitative data
Closed and fuzzy closed mining	Closed FIM; fuzzy closed itemset mining^17^^18^,	Redundancy reduction with support preservation	Can still generate many closed itemsets in dense datasets and does not target maximal compactness
Fuzzy FP-Growth	Adaptive FP-Growth; temporal fuzzy FP-Growth^19^^20^,	Efficient fuzzy or temporal frequent pattern mining	Improves mining efficiency but generally focuses on frequent pattern discovery rather than maximal fuzzy compactness
Type-2 fuzzy mining	T2FM^21^	Type-2 fuzzy frequent itemset mining	Models fuzzy uncertainty but does not directly target maximal fuzzy pattern extraction
Top-k frequent itemset mining	Top-k frequent itemset methods^22^	Mining top-ranked frequent itemsets	Not designed for fuzzy linguistic representation or maximal fuzzy pattern extraction
Distributed and rule mining	Distributed ARM; P-FARM^23^^25^,	Distributed or profit-oriented rule extraction	Produces association rules rather than compact MF′Ps
Graph-based mining	Maximal biclique discovery^24^	Graph or bipartite pattern structures	Uses a different data model and output semantics from transactional fuzzy itemset mining
Proposed MFPM	MFPM	MF′Ps from quantitative data	Integrates fuzzy linguistic transformation, maximum-cardinality pruning, ternary longest-pattern discovery, and Anti-Apriori maximal extraction

Previous research in fuzzy pattern mining reveals several methodological gaps. Studies by Wu et al. and Kumar et al. successfully integrated fuzzy theory with pattern mining but maintained conventional enumeration approaches, consequently inheriting the pattern explosion problem. The parallel framework developed by Wu et al. addressed scalability through distributed computing but did not resolve the fundamental issue of output complexity. Similarly, while Cui et al. expanded mining to rare itemsets, their methodology continued to generate complete pattern sets without redundancy elimination. Research in maximal pattern mining, such as the graph-based approaches of Nguyen et al. and sequential methods of Li et al., demonstrated the effectiveness of maximal representations for binary and temporal data. However, these studies did not extend their methodologies to handle the specific challenges of quantitative data through fuzzy set integration. The theoretical work on fuzzy algebraic structures by Mahalakshmi et al. and Kengne et al. laid important foundations for facilitating maximality in fuzzy setups, but had limited to no deliverable mining implementation for real-world datasets. Zhang et al., importantly and uniquely, focus on seasonal-periodic subgraphs, offering insight into how to incorporate temporal sensitivity. However, their σ-periodic Ω-seasonal model is orthogonal to fuzzy quantitative semantics and does not limit redundancy among more frequent variants overall. Fournier-Viger’s notion of local periodic patterns, Wu’s OPP-Miner supports temporal ordering, while Li’s MCoR-Miner suggests pruning overlapping, sequential rules. However, these sequences retain crisp attributes with limited arrangements of graded membership or maximal fuzzy consolidation. Similarly, other theoretical works on fuzzy algebra (e.g., hesitant or Δ-open sets, fuzzy ideals, and granular matrices) suggest an incrementally deeper notion of extremal terms of maximality. Still, they would not yield implementable pipelines for dense transactional data.

A common limitation in previous work was that no integrated methodology was proposed to account for quantitative data uncertainty using fuzzy sets, and simultaneously utilize maximal pattern representation. By providing a maximal representation instead of enumerating each pattern, as done in previous work, the MFPM method represents a critical advancement, reducing patterns’ density by approximately 94.97%, and subsequently achieving gains using fuzzy mining compared to binary maximal mining. In addition, although we are unaware of any binary maximal mining works that incorporate fuzzy set theory, we demonstrate that fuzzy set theory handles quantitative data reasonably effectively. Even though thicker, dominant variables also have fuzzy set representations, the MFPM framework shows the superior performance of subset pattern premises over FTDA, while achieving greater than 65% computational efficiency in lower support difficulties. Moreover, we produced semantically inter-represented patterns, demonstrating the shape or linear concept needed to inform patterns that are part of the relationship of knowledge. Each component of the framework made methodological contributions, including integrated maximal fuzzy pattern mining, aggressive multi-stage pruning approaches, logarithmic-length pattern search through ternary search methods, and the handling of zero values. Together, the contributions lead to scalable quantitative dataset mining, producing compressed, non-redundant pattern sets that enhance decision-making capabilities in other applications. Empirically, significant pattern compressions and runtime reductions were observed in other dense benchmarks, while retaining linguistic interpretability. In doing so, MFPM closes the gap between semantic fidelity and computational tractability left open by temporal, utility, rare-itemset, and graph-centric methods.

Key strengths: (1) semantically faithful fuzzification (with explicit handling of zeros) to avoid sharp boundaries; (2) principled, multi-stage pruning (maximum-cardinality, early termination, superset dominance) anchored in fuzzy anti-monotonicity; (3) directed, logarithmic longest-length discovery; (4) compact, maximal fuzzy outputs that eliminate redundancy and enhance analyst focus. The result is a scalable, domain-agnostic pipeline delivering fewer, more meaningful patterns.

Additionally, the current implementation focuses on static datasets, which limits its direct applicability to streaming data environments.

## Practical applications of the proposed MFPM framework

The proposed MFPM framework is suitable for fields where data are quantitative, dense, and uncertain, making them difficult to interpret with precise pattern-extraction methods. These conditions are common in many practical applications, where rigid segmentation can lead to sharp boundary problems, and traditional repetitive pattern extraction can produce over-interpretations.

In healthcare analytics, MFPM can support the discovery of interpretable relationships between clinical variables, such as physiological measurements, laboratory indicators, glucose levels, blood pressure, cholesterol levels, or clinical data derived from sensors. Fuzzy terminology, such as “low,” “medium,” and “high,” can improve interpretability for clinicians by representing gradual shifts rather than rigid time intervals. The pattern compression observed in the Mushroom dataset results in Sect. 4.3 illustrates the type of reduction that can be useful in high-dimensional clinical feature spaces.

In business intelligence, MFPM can help identify meaningful correlations between customer and transactional characteristics, such as purchase frequency, spending level, product quantity, and customer value. By extracting the most frequent fuzzy patterns, this framework provides concise behavioral summaries. Table 11 illustrates how patterns are reduced in MFPM and how it can distill large outputs into fewer, more actionable analytical patterns.

In IoT and smart environments, MFPM can be applied to sensor data, including temperature, humidity, power consumption, device activity, and other environmental measurements. This data is often continuous, fuzzy, and uncertain. Fuzzy modeling captures incremental changes, while maximal pattern extraction identifies prevailing co-occurrence relationships.

In cybersecurity, MFPM can support anomaly detection and intrusion behavior analysis using quantum network properties, such as packet rate, session duration, traffic volume, login frequency, and network interaction intensity.

## Conclusions

This study has successfully established an efficient framework for mining MF′Ps from dense quantitative datasets, effectively addressing the critical challenge of pattern explosion that has limited the practical application of traditional fuzzy pattern mining approaches. The integrated MFPM methodology demonstrates that combining fuzzy set theory with maximal pattern representation can achieve substantial computational advantages while preserving essential knowledge relationships. Through systematic evaluation across multiple benchmark datasets, the framework has demonstrated its capability to generate compact pattern sets, reducing output volume by up to 94.97% compared to conventional approaches, while also improving computational efficiency by over 65% in low-support scenarios. The implementation of innovative strategies—including maximum cardinality pruning, ternary search for longest pattern identification, and Anti-Apriori traversal with superset pruning—has demonstrated significant improvements in both scalability and interpretability. The discovered patterns maintain semantic richness through fuzzy linguistic representations while eliminating redundancy, providing domain experts with actionable insights without overwhelming analytical capacity. The study contributions advance the field of quantitative data mining by providing a mathematically rigorous yet practical solution to the pattern explosion problem, establishing a foundation for further developments in maximal fuzzy pattern mining.

Although the experimental results demonstrate promising improvements in pattern reduction and computational efficiency, two future extensions can further strengthen the framework.

This study evaluates MFPM under a controlled experimental setting using benchmark datasets. This design ensures reproducibility and enables fair comparison with existing methods. Future work will extend the evaluation to domain-specific quantitative datasets, such as healthcare, business analytics, IoT, and cybersecurity, to further examine the framework’s effectiveness in applied environments.

## Data Availability

Data availability: Data Availability: The original benchmark datasets used in this study—Chess, Connect, and Mushroom—are publicly available through the preserved Frequent Itemset Mining Dataset Repository at: https://www.cs.rpi.edu/~zaki/Workshops/FIMI/data/The datasets can also be accessed directly through the following links: Chess: https://www.cs.rpi.edu/~zaki/Workshops/FIMI/data/chess.dat.gzConnect: https://www.cs.rpi.edu/~zaki/Workshops/FIMI/data/connect.dat.gzMushroom: https://www.cs.rpi.edu/~zaki/Workshops/FIMI/data/mushroom.dat.gz.
